# Homogeneous and heterogeneous catalytic reduction of amides and related compounds using molecular hydrogen

**DOI:** 10.1038/s41467-020-17588-5

**Published:** 2020-08-04

**Authors:** Jose R. Cabrero-Antonino, Rosa Adam, Veronica Papa, Matthias Beller

**Affiliations:** 1 Instituto de Tecnología Química. Universitat Politècnica de València - Consejo Superior Investigaciones Científicas (UPV-CSIC). Avda. de los Naranjos s/n, València, 46022 Spain; 2 Leibniz-Institut für Katalyse, Albert-Einstein-Strasse 29a, Rostock, 18059 Germany

**Keywords:** Catalysis, Homogeneous catalysis

## Abstract

Catalytic hydrogenation of amides is of great interest for chemists working in organic synthesis, as the resulting amines are widely featured in natural products, drugs, agrochemicals, dyes, etc. Compared to traditional reduction of amides using (over)stoichiometric reductants, the direct hydrogenation of amides using molecular hydrogen represents a greener approach. Furthermore, amide hydrogenation is a highly versatile transformation, since not only higher amines (obtained by C–O cleavage), but also lower amines and alcohols, or amino alcohols (obtained by C–N cleavage) can be selectively accessed by fine tuning of reaction conditions. This review describes the most recent advances in the area of amide hydrogenation using H_2_ exclusively and molecularly defined homogeneous as well as nano-structured heterogeneous catalysts, with a special focus on catalyst development and synthetic applications.

## Abstract (web summary)

Catalytic hydrogenation of amides is a pivotal chemical transformation for both research labs and chemical production in industry. Here, the authors comprehensively review this topic by including state-of-art homogeneous and heterogeneous catalysts that can hydrogenate amides and related compounds.

## Introduction

The development of green and sustainable methods is a central focus of modern organic synthesis and its applications in various areas of the chemical industry. In this respect, many reduction reactions, traditionally employing stoichiometric amounts of metal hydrides with inherent low atom efficiency^1^, can be favorably replaced by catalytic hydrogenations. In fact, the combination of molecular hydrogen and catalysts does not only allow avoiding formation of waste products^2^, it also opens the door to clean transformations based on renewable energies as the conversion of solar or wind energy to “green” hydrogen is likely to be implemented in the coming years. Among the many reduction reactions known, amide hydrogenation can be encountered as one of the most desired considering the importance of the derived amines in several branches of chemical industry. In fact, since the start of the Green Chemistry Institute Pharmaceutical Roundtable in the United States in 2005, several projects aimed to the achievement of a practical amide hydrogenation methodology have been supported^3,4^. The long-term interest of the pharmaceutical industry in this specific transformation is explained by the fact that it ideally affords structurally complex amines, present in many of the currently employed drugs. Furthermore, amines are also in bulk and fine chemicals used in the manufacture of plastics, textiles, surfactants, dyes, and many more^5^.

Amides play a central role in the chemistry of life, as they are the key elements to generate peptides and proteins from amino acid monomers. At the same time, they are present in artificial polymeric materials such as nylon or polyacrylamides produced on multi-million-ton scale. Hence, organic chemists developed, in the past century, literally a myriad of methodologies for their synthesis^6^. Their ubiquity derives from their stability conferred by their chemical structure, being the most stable carboxylic acid derivatives as they are least reactive mainly toward the nucleophilic attack at the carbonyl group^7–9^. Amides inertness is mainly caused by the different electronic character of nitrogen and oxygen in their structure^10^. This leads to resonance stabilization, rendering the carbonyl group less electrophilic than the carbonyl of other carboxylic acid derivatives (see Box 1). Taking into account this, amides with a lesser electron density at the carbonyl group such as N-aryl amides, formamides and, as even more extreme cases, trifluoromethyl acetamides or amides containing fluorinated or CF_3_-substituted aryl groups, can be considered relatively activated amides toward hydrogenation.

Despite the difficulties encountered for the hydrogenation of amides, continuous efforts are reported in this field. In this review, we focus on the different strategies that both in homogeneous and heterogeneous catalysis have been described to overcome this problem. In addition, selected recent applications of interest for organic synthesis or alternative energy technologies, in which an amide bond hydrogenation is an essential step, are also commented. Finally, we present our point of view about the future perspectives of this field of research.

Box 1. Structural properties of the amide group determining its stability

The difference of electronegativity between oxygen and nitrogen in amides leads to a delocalization of the nitrogen electronic lone pair. Hence, it is possible to express the amide group as a resonant form between neutral and zwitterionic configurations^171^. The electronic contribution of nitrogen makes the carbonyl functional group less activated toward the attack of hydride or other nucleophiles. This effect is influenced by the electronic character of the nitrogen substituents and is only possible in amides in which the C–O and C–N bonds are in the same plane. The contribution of both configurations justifies some of the observed properties in amides such as the partial double bond character of C–N bond or the low basicity of nitrogen^172^.

Primary and secondary amides present an equilibrium in solution with their enol tautomers, clearly disfavored thermodynamically for the iminol^173,174^, albeit possibly presenting some kinetic relevance in the hydrogenation mechanism.

Intermolecular H bonds of primary and secondary amides play a main role determining the secondary structure of proteins^175^. These intermolecular forces, which can also be established between amides and/or iminols and protic solvents^176^, also contribute to the higher resistance of these carboxylic acid derivatives to hydrogenation.

## History of amide hydrogenation and selectivity issues

Owing to the ease of amide formation and the general availability of amides, their catalytic hydrogenation offers interesting perspectives for the synthesis of structurally complex amines^8,9,11–16^. Nevertheless, traditional processes such as reductive amination, hydrogenation of nitro compounds, or nitriles are prevailing in industry and organic synthesis^17–19^. In addition, amines have also been accessed in a very chemoselective manner through catalytic amide reduction protocols using hydrosilanes or hydroboranes as reducing agents^11–13,20,21^. However, these methodologies suffer from a sustainability perspective when compared with catalytic hydrogenation owing to their high level of waste production, hence are not suitable methods for industrial applications.

**Fig. 1:**
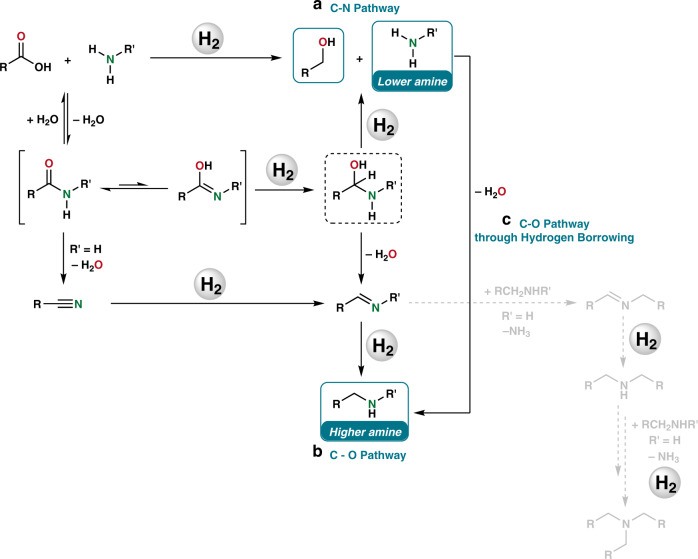
Mechanisms proposed for amides hydrogenation. **a** C–N through hemiaminal or carboxylic acid intermediates; **b** C–O through hemiaminal or nitrile intermediates, (in gray byproducts formation from imine); **c** C–O through hydrogen borrowing.

Original reports for catalytic hydrogenation of amides employed heterogeneous materials at harsh reaction conditions (H_2_ pressures 200–300 bar, temperatures >200 °C). In the past two decades, there is a growing interest among chemists in developing feasible catalysts working at milder conditions. Besides, a second major challenge is the control of the selectivity (N- vs O-hydrogenolysis). As it is shown in Fig. 1, the main reaction products that can be obtained from amide hydrogenation are either the mixture of an alcohol and the lower amine (Fig. 1a), resulting from the C–N hydrogenolysis, or the higher amine (Fig. 1b), derived from the C–O pathway. This latter pathway is more attractive for most synthetic applications. However, for certain industrial applications (e.g., methanol), or in intramolecular cases (e.g., lactams), the C–N cleavage can be desired, too. Finally, it offers interesting possibilities for deprotection methodologies.

The main mechanistic proposal for both pathways consists of a first addition of hydrogen to the amide, or its tautomer iminol, to afford the corresponding hemiaminal intermediate^22^ that can undergo hydrogenolysis giving the alcohol plus the lower amine, in the case of the C–N pathway (Fig. 1a). Alternatively, the hemiaminal can be dehydrated to give an imine that further hydrogenates to the higher amine (C–O pathway, Fig. 1b). Interestingly, in the case of tertiary amides bearing a hydrogen in α to the carbonyl, an enamine intermediate^20^ can be formed and, therefore, the C–O hydrogenation of this kind of amides is, for some catalytic systems^23–25^, relatively favored in comparison with the tertiary amides which only can give the iminium intermediate. It should be noted that other side-products, such as secondary and tertiary amines and imines have been observed in amides hydrogenation (gray part of Fig. 1b), too^26^. This is an indirect proof of the existence of imine intermediates in the C–O cleavage pathway. It has also been proposed that these products can be formed by the transamidation of the starting amide with the higher amine followed by hydrogenation^27^. In addition to the generally accepted mechanism involving hemiaminal formation, some alternatives have been suggested based on indirect observations: for example, the hydrolysis of the amide to a carboxylic acid that is subsequently hydrogenated to the corresponding alcohol^8^, or the dehydration of the amide (preferentially primary) to a nitrile that is further hydrogenated to the amine^28,29^. Recently, another pathway has been identified consisting in a formal C–O hydrogenation, which starts with a regular C–N cleavage to give an alcohol and an amine, which undergo a hydrogen borrowing (HB) mechanism to give the higher amine (Fig. 1c)^30^.

Classically, the C–O cleavage pathway is prevailing in the presence of bimetallic heterogeneous catalysts or, to a lesser extent, with homogeneous catalysts under acidic conditions, whereas C–N cleavage is known to proceed using homogeneous catalysts in basic media. In fact, in an interesting discrete Fourier transform (DFT) study performed in 2011 for molecularly defined [Ru-PNN] complexes affording C–N cleavage, it was calculated that this pathway was 10 kcal mol^−1^ lower in energy than the C–O pathway^31^.

## Development of homogeneous catalysts

The last decade has been a crucial period in the design and development of tailor-made complexes for the hydrogenation of amides either through C–N, or C–O pathway. Metal-ligand cooperation strategy and the employment of specific ligands in combination with suitable acidic additives have been key points to achieve novel active systems for the hydrogenation of amides.

### Lower amines and alcohols or amino alcohols by C–N hydrogenation

**Fig. 2:**
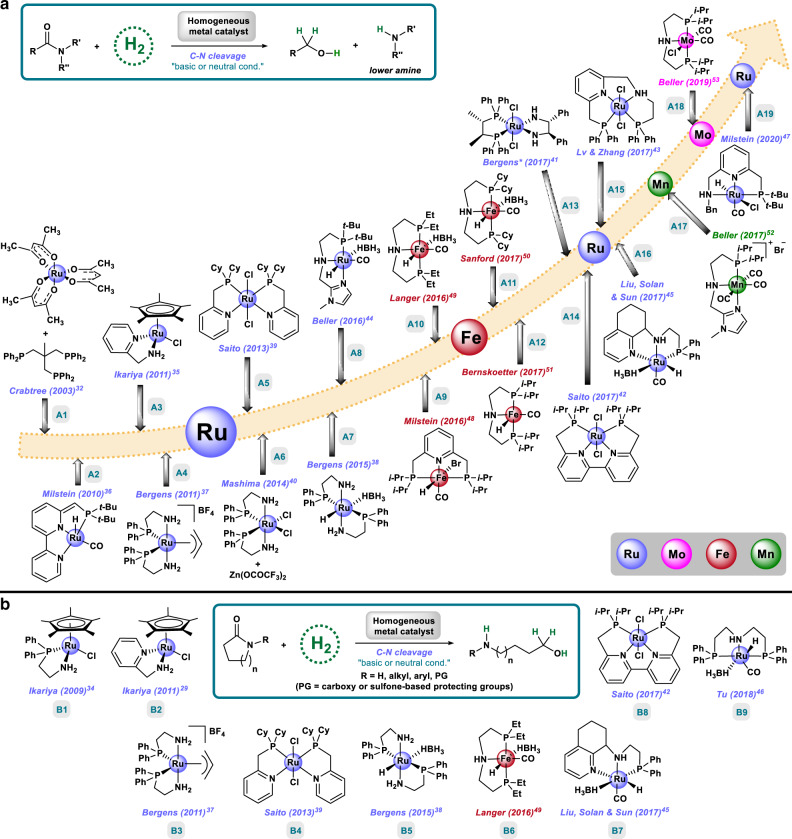
Evolution of amide hydrogenation catalysts. **a** Homogeneous catalytic systems for hydrogenation of amides to alcohols and amines by selective C–N hydrogenolysis, from ruthenium to iron, manganese, and molybdenum. (*=enantioselective protocol). **b** Molecularly defined complexes performing lactams hydrogenation to amino alcohols by C–N cleavage.

In 2003, the first homogeneous deaminative hydrogenation of amides was reported in a patent dealing with the hydrogenation of carboxylic acid derivatives employing a [Ru/Triphos] catalyst system (Fig. 2A1)^32^. Besides this early work, more recently reported homogeneous catalysts active for the hydrogenation of amides by C–N cleavage operate through a metal-ligand cooperation strategy using various ruthenium bi- or tridentate complexes^33^. Similar to the bond activation mechanism followed in enzymes, in these systems a finely adjusted ligand environment (having NN, PN, PNP, or PNN donor atoms) is in cooperation with the metal center, which enables heterolytic hydrogen activation, reduction to the hemiaminal, and finally C–N cleavage. Thus, there is no overall change of oxidation state of the metal and the hydrogen activation occurs either across metal–ligand bond (frequently M–N) or through disruption/restoration of the aromaticity of the ligand. Most of these complexes need basic conditions to operate, as they constitute pre-catalysts, being the active catalyst a deprotonated species such as an amido complex (M=N) or a dearomatised complex. In a similar manner, basic additives might also facilitate catalyst regeneration from the stable resting state.

In 2009, Ikariya and co-workers described the first hydrogenation of N-protected lactams to N-protected amino alcohols using a [Ru-PN] bidentate complex and stoichiometric amounts of KO*t*-Bu, where the Ru–NH has a major role in hydrogen activation (Fig. 2B1)^34^. Two years later, the same group reported an improved complex, also bearing a Ru–NH functionality, which affords the hydrogenation of unprotected lactams and two amides, using catalytic amounts of base (Fig. 2A3 and B2)^35^. The first pincer complex able to hydrogenate a broad scope of secondary and tertiary amides at 110 °C meant an important breakthrough in the field and it was reported by Milstein group in 2010 (Fig. 2A2)^36^. In this case, the reaction can be performed either in the absence of base, when using the dearomatised complex, or with one equivalent of base with respect to Ru, when employing the more stable aromatic pre-catalyst. In addition, complexes bearing two [PN] bidentate ligands, similar to Noyori’s hydrogenation catalyst, have also been explored for this reaction by the groups of Bergens^37,38^, Saito^39^, and Mashima^40^ affording the synthesis of alcohols and amines or amino alcohols in good yields (Fig. 2A4–7, B3–5). In 2017, the group of Bergens achieved the first enantioselective deaminative hydrogenation of α-phenoxy tertiary amides by dynamic kinetic resolution, using a chiral derivative of Noyori-type complex in the presence of (over)stoichiometric amounts of 2-PrONa and 2-PrOH (Fig. 2A13)^41^. With the aim of improving catalyst stability and, hence, paving the way for having access to challenging substrates, the same year the groups of Saito^42^ and Lv & Zhang^43^ published two tetradentate complexes with [Ru-PNNP] structure (Fig. 2A14–15 and B8) active in the presence of catalytic amounts of base. A remarkable substrate scope including primary (one example), secondary, and tertiary amides including caprolactame and polyamides was achieved using Saito’s complex, that also showed chemoselectivity toward the hydrogenation of the amide group in the presence of alkenes. Around the same time, the groups of Beller^44^ and Liu, Solan, and Sun^45^ reported two [Ru-PNN] pincer complexes able to hydrogenate primary, secondary, and tertiary amides in base-free conditions (Fig. 2A8, A16, and B7). Both complexes present a Ru–BH_4_ moiety, being able to deliver the active catalyst at the reaction conditions. In 2018, Tu et al.^46^ showed that the commercially available [Ru-MACHO-BH] complex is an active catalyst for the synthesis of protected and unprotected amino alcohols by deaminative hydrogenation of lactams employing catalytic amounts of K_3_PO_4_ (Fig. 2B9). Finally, this year Milstein and co-workers reported a [Ru-PNN] pincer complex able to hydrogenate secondary and tertiary amides in the presence of catalytic amounts of base at extremely mild conditions (room temperature and 5–10 bar of H_2_) and performing via a Ru–N amido intermediate (Fig. 2A19)^47^.

Since the new millennium, in homogeneous catalysis there is a strong interest to develop new defined catalysts based on non-precious metals. The principle of metal-ligand cooperation offers a great opportunity in this direction. Apart from other 3d metals, especially iron and manganese are interesting owing to their low toxicity and high natural abundance. In 2016, pioneering work in this area was reported by Milstein and co-workers describing the first iron catalyst able to mediate the C–N hydrogenation of activated amides (mainly trifluoroacetamides) (Fig. 2A9)^48^. Here, the pre-catalyst was a [Fe-PNP] complex that needed catalytic amounts of KHMDS (potassium bis(trimethylsilyl)amide) to generate the active dearomatised complex. After that, the groups of Langer^49^, Sanford^50^, and Bernskoetter^51^ reported three [Fe-PNP] complexes, with a very similar structure, and able to mediate the deaminative hydrogenation of secondary and tertiary amides in base-free conditions (Fig. 2A10–12 and B6). In 2017, Beller and co-workers reported the first example of a manganese based complex able to catalyze the C–N cleavage of 1°, 2° (including diflufenican), and 3° amides as well as formamides, showing chemoselectivity toward the hydrogenation of the amide group in the presence of carbamates and ureas (Fig. 2A17)^52^. More recently, the same group described a family of molybdenum pincer complexes selective for the C–N hydrogenolysis of N-methyl formanilides owing to the catalyst deactivation in the presence of alcohols or secondary amides (Fig. 2A18)^53^.

### Higher amines by C–O hydrogenation

Compared with the deaminative process, the deoxygenative hydrogenation of amides produces the corresponding higher amines and H_2_O. From a synthetic point of view, this methodology is more interesting as it allows in principle the efficient formation of new C–N bonds via N-acylation and subsequent hydrogenation^54^. For these reasons, in 2005 the ACS Green Chemistry Institute and members of the so-called Pharmaceutical Roundtable named this transformation as a “dream reaction” for future developments in process chemistry^3,4^. Originally, this reaction has been performed employing heterogeneous catalysts; however, in the last decade several homogeneous organometallic complexes became of interest and were optimized for the C–O hydrogenation of amides. Many of these latter systems are based on Ru complexes with the specific phosphine ligand: 1,1,1-tris(diphenylphosphinomethyl)ethane (so-called Triphos). Notably, most of these homogeneous catalysts need the presence of acid additives to be selective for deoxygenative hydrogenation. In fact, in the last years, several examples using a variety of catalysts in the presence of boron-based Lewis acid additives have appeared. In general, these additives are thought to promote the C–O type hydrogenation by Lewis acidic activation of the amide and subsequent dehydration. Until very recently, for all these transformations the same mechanism, normally proposed for the C–O hydrogenation of amides, was assumed (Fig. 1, path b). Interestingly, in 2016 our group demonstrated^30^ for a [Ru/Triphos] type system that a C–N cleavage/amine N-alkylation through alcohol HB mechanism is operating as main reaction pathway (Fig. 1, path c). This kind of mechanism had been previously suggested by Saito and co-workers for the reduction of lactams^39^. Understanding in detail the mechanism by which the different catalytic systems operate is a relevant point in order to rationalize the results and, what is more important, be able to design improved systems. Therefore, we will emphasize the most probable reaction mechanism for each example shown below, when possible.

**Fig. 3:**
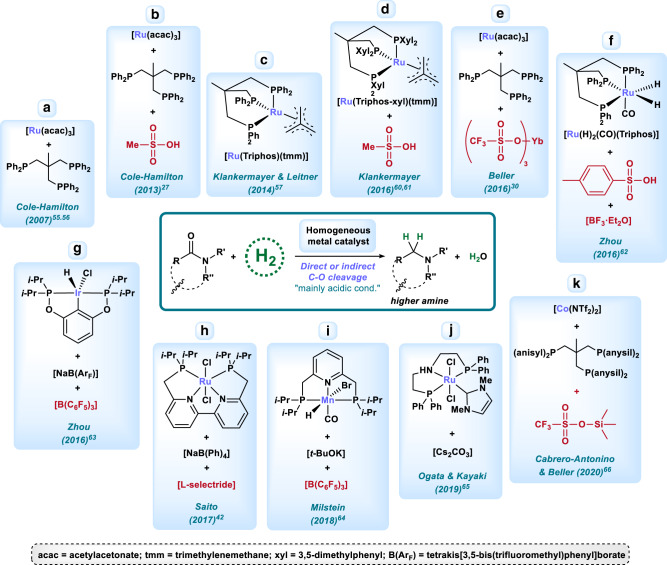
Deoxygenative hydrogenation. Schematic representation of homogeneously catalyzed deoxygenative hydrogenation of amides to amines and the corresponding molecular catalysts used for the process.

Early examples of homogeneous hydrogenations involving C–O cleavage of amides were reported by Cole-Hamilton and co-workers using a combination of [Ru(acac)_3_] and Triphos as catalyst system (Fig. 3a)^55,56^. Later on, the same authors, together with the group of Leitner and Klankermayer, recognized serious reproducibility issues for this system. Gratifyingly, in 2013 they discovered that the former system becomes more reproducible and general by addition of methanesulfonic acid (MSA) (Fig. 3b)^27^. Using such combination, a broad range of secondary and tertiary amides could be hydrogenated in moderate to good yields, albeit at temperatures up to 220 °C. In their work, the authors assumed a classical C–O cleavage pathway, explaining the formation of the byproducts through transamidation processes^27^. One year later, the same groups presented an in-depth study to understand the active intermediate species relevant in this system (Fig. 3c)^57^. The authors established, by spectroscopic investigations and DFT calculations, that the presence of the acid additive is in many cases required to avoid catalyst deactivation mainly through the formation of a dimeric-hydride^58^, as well as carbonyl-containing complexes^59^.

Apart from the co-catalyst, the tridentade ligand Triphos has a key role in deoxygenative amide hydrogenations. In 2016, to understand the peculiar ligand behavior, Klankermayer and co-workers synthetized the related well-defined complex [Ru(Triphos-xyl)(tmm)] featuring a 3,5-dimethylphenyl-substituted Triphos ligand (Fig. 3d)^60^. This novel complex, in combination with MSA as additive, showed enhanced activity in lactam hydrogenations (at 160 °C and 100 bar of H_2_) to the corresponding cyclic amines^60^. In the same year, our group presented the combination of [Ru(acac)_3_/Triphos/Yb(OTf)_3_] as an improved catalyst system for the deoxygenative hydrogenation of >25 amides under milder conditions (150 °C and 5 bar of H_2_) (Fig. 3e)^30^. As it was commented before, in a set of control experiments and kinetic studies, it was shown that the higher amine product is actually formed by an initial C–N hydrogenolysis that affords an alcohol and a lower amine, which is a common pathway for homogeneous catalysts. Then, the lower amine undergoes N-alkylation with the so-formed alcohol through a HB or autotransfer mechanism, affording finally the higher amine (Fig. 1, path c)^30^. At this point, it should be considered that the previous reported examples from the groups of Cole-Hamilton, Leitner and Klankermayer could be also operating through this mechanism, although an empirical demonstration for that specific reaction conditions is still missing. On the other hand, such two-step mechanism was further confirmed and applied by Klankermayer and co-workers for the synthesis of cyclic tertiary amines from lactams and alcohols in the presence of [Ru(Triphos-xyl)(tmm)] complex and MSA (Fig. 3d)^61^. Moreover, the authors showed that the lactam can be hydrogenate/N-alkylated even with the absence of molecular hydrogen, just by using an alcohol as hydrogen transfer reagent^61^. Almost at the same time, the group of Zhou and co-workers reported the selective hydrogenation of secondary amides by C–O cleavage using the [Ru(H)_2_(CO)(Triphos)] complex as catalyst in the presence of catalytic amounts of TsOH and BF_3_·Et_2_O at 120 °C (Fig. 3f)^62^. The authors proposed that the presence of a boron-based Lewis additive (BF_3_·Et_2_O) directly activates the amide oxygen atom affording an amide-boron adduct as key intermediate. However, whether the reaction proceeds through the dehydration of the resulting hemiaminal or via C–N cleavage was not investigated.

In addition, the same group developed a different system consisting of the well-defined Ir-[P(O)C(O)P] complex able to catalyze the deoxygenative hydrogenation of a wide range of secondary amides in the presence of stoichiometric amounts of [B(C_6_F_5_)_3_] and catalytic amounts of [NaBAr_F_] as additives at 120 °C (Fig. 3g)^63^. The authors proposed that in this system there is an initial formation of an Ir-H complex through chloride exchange by [NaBAr_F_] and hydrogenation, followed by a direct C–O cleavage mechanism where [B(C_6_F_5_)_3_] activates the amide and promotes the dehydration step. Although a direct C–O cleavage in the presence of stoichiometric amounts of [B(C_6_F_5_)_3_] seems reasonable, no experimental evidences were provided. Similarly, in 2017, the Saito group showed two examples of deoxygenative hydrogenation of ε-caprolactames to azepanes by using a [Ru-PNNP] complex with catalytic amounts of two boron additives (NaB(Ph)_4_ and *L*-selectride) (Fig. 3h)^42^. In contrast, in the same work the [Ru-PNNP] demonstrated to be an effective catalyst for the C–N hydrogenation of various amides when NaH was used as additive, pointing to the large influence of the boron additives on the selectivity. The authors proposed that the reaction for the two lactams can be occurring either via direct C–O hydrogenation or C–N hydrogenolysis/N-alkylation H-borrowing route.

In 2018, an interesting advancement was reported by Milstein group, who achieved the first C–O hydrogenation of amides with a molecularly defined base metal catalyst (Fig. 3i)^64^. Explicitly, a [Mn-PNP] pincer complex in the presence of catalytic amounts of KO*t*-Bu and stoichiometric amounts of [B(C_6_F_5_)_3_] hydrogenated different secondary amides to the desired amines at relatively severe reaction conditions (50 bar of H_2_, 150 °C, 72 h). Last year, Ogata & Kayaki and co-workers reported a formal deoxygenative hydrogenation of lactams, including a wide range of unprotected substrates, with a [Ru-PNP] complex in the presence of Cs_2_CO_3_ (0.5–1 eq.), under 30–50 bar of H_2_ at 120–150 °C (Fig. 3j)^65^. In this work, the authors demonstrate that the system works through a C–N hydrogenolysis/HB mechanism. These two examples are the only homogeneous systems operating via metal–ligand cooperation in the presence of base, leading to a C–O hydrogenation of amides.

Following the general interest in non-noble metal catalysis, the first cobalt-catalyzed deoxygenative hydrogenation of amides to amines was recently developed by Cabrero-Antonino & Beller and co-workers (Fig. 3k)^66^. The catalytic system consists of [Co(NTf_2_)_2_] and (*p*-methoxyphenyl)triphos in the presence of [Me_3_SiOTf] as acidic co-catalyst, allowing the direct hydrogenation of amides to the corresponding amines under milder conditions (125 °C; 1–10 bar H_2_). A set of control experiments indicated that the reaction mainly proceeds through C–O bond cleavage, though other minor pathways might be involved.

Finally, Grimme and Paradies reported very recently the C–O hydrogenation of tertiary amides employing a metal-free frustrated Lewis pair catalyst in the presence of over-stoichiometric amounts of oxalyl chloride^67^.

## Development of heterogeneous catalysts

Pioneering examples in the field of amide hydrogenation employed heterogeneous catalysts at harsh reaction conditions affording mainly the corresponding amines through C–O cleavage reaction. In fact, the standard catalytic system for hydrogenating an amide until the mid of the 80’s was the [Cu/CrO] catalyst^68^, developed by Adkins at the beginning of the century. Unfortunately, this catalyst requires high temperatures (∼250 °C) and hydrogen pressures between 200 and 300 bar. Other alternatives, less studied, are Ni Raney^69^ or Re black^70^ that also need similar reaction conditions. Apart from the obvious drawback of drastic conditions, these systems also have serious selectivity problems, usually solved by the employment of ammonia type additives.

Introducing the concept of bifunctional catalysis, allowed for a major step forward in the field in terms of milder reaction conditions and improved selectivity. This concept is based on the idea of combining a transition metal from the groups 6 or 7, presenting an oxophilic character and, hence, able to activate the carbonyl group, with another from groups 8 to 10, widely known by their efficient activation of hydrogen. Early examples of this concept are the combination of Pd/Re supported on high surface area graphite/zeolite 4 Å published in a BP patent in 1988^71^, and the work of Fuchikami et al.^26^ in which several Rh and Ru precursors were combined with Re, Mo, and W species, in principle either of homogeneous or heterogeneous nature. In the last two decades, this concept has been studied first by Whyman and co-workers who between 2010 and 2011 published a series of three papers rationalizing the concept of bifunctional unsupported catalysts for the hydrogenation of amides (Table 1, entries 1–4)^28,29,72^. Whyman et al. performed the hydrogenation of mainly aliphatic amides with a series of unsupported bimetallic heterogeneous catalysts of Rh or Ru with Mo or Re, formed in situ at the reaction media from their metal carbonyl precursors. Through the employment of IR, X-ray powder diffraction (XRD), transmission electron microscopy, energy dispersive X-ray, and X-ray photoelectron spectroscopy techniques, they rationalized that the active material was composed by particles of Rh(0) or Ru(0) of 2–4 nm surrounded by Mo or Re in different oxidation states. Key for the formation of an active material is the use of the optimum proportion between the two metals to achieve an intimate contact between Rh or Ru and Mo or Re. The amide order of reactivity for these catalysts is 1°>3°>>2°, being very selective to C–O cleavage and without the need of ammonia as additive to avoid the formation of alkylated amines. This preferential reactivity for primary amides led the authors to propose a possible nitrile type mechanism implying dehydration, specially favored for the hydrogenation of primary amides, although there are no experimental evidences of this behavior.

**Table 1:** Heterogeneous catalytic hydrogenations of amides developed over the last decade.

Entry	[Catalyst]	Amide type (n° ex.)	Scission pathway	Conditions	Ref.
1	[Rh_6_(CO)_16_/Mo_2_(CO)_6_] (1:0.6)	1° (3), 2° (1), 3° (1)	C–O	130–160 °C, 50–100 bar H_2_, DME	^72^
2	[Ru_3_(CO)_12_/Mo_2_(CO)_6_] (1:0.5)	1° (4), 2° (2), 3° (4)	C–O	145–160 °C, 20–100 bar H_2_, DME	^29^
3	[Ru_3_(CO)_12_/Re_2_(CO)_10_] (1:1)	1° (1), 3° (1)	C–O	160 °C, 100 bar H_2_, DME	^28^
4	[Rh_6_(CO)_16_/Re_2_(CO)_10_] (1:1)	1° (1), 3° (1)	C–O	160–180 °C, 100 bar H_2_, DME	^28^
5	[PtRe/TiO_2_]	2° (1), 3° (1)	C–O	120 °C, 20 bar H_2_, hexane	^73,74^
6	[PdRe/Graphite]	2° (50), 3° (58)	C–O	120–160 °C, 10–30 bar H_2_, DME	^25^
7	[Rh-MoO_x_/SiO_2_ + CeO_2_]	1° (1)	C–O	120 °C, 80 bar H_2_, DME	^78^
8	[Ni/LaAlSiO]	3° (1)	C–O	150 °C, 40 bar H_2_, DME/Ethylene glycol	^76^
9	[Pt/Nb_2_O_5_]	3° (12)	C–O	160–200 °C, 50 bar H_2_, neat	^77^
10	[PtV/HAP]	2° (3), 3° (19)	C–O	RT-70 °C, 1–5 bar H_2_, DME	^23^
11	[Re/TiO_2_]	2° (4), 3° (8)	C–O	180–200 °C, 50 bar H_2_, octane	^24^
12	[Ir/3Mo-KIT-6]	3° (1)	C–O	130 °C, 30 bar H_2_, DME	^75^
13	[RuWO_x_/MgAl_2_O_4_]	1° (6), 2° (1)	C–O	200 °C, 50 bar H_2_, 6 bar NH_3_, CPME	^79^
14	[Ag/γ-Al_2_O_3_]	1° (1), 2° (12), 3° (1)	C–N	150 °C, 50 bar H_2_, KOt-Bu, 1,4-dioxane	^81^
15	[Ru/CeO_2_]	1° (8), 2° (1), 3° (1)	C–N	60 °C, 80 bar H_2_, water	^82^
16	[Pd/In_2_O_3_]	1° (3), 2° (14), 3° (5)	C–N	160 °C, 60 bar H_2_, toluene	^83^

Once the feasibility of bifunctional catalysis for the amides hydrogenation was demonstrated, it was desirable to design supported catalysts, whose structure can be more easily controlled. Furthermore, two main points still needed to be fulfilled: the hydrogenation of secondary amides and the selectivity toward the hydrogenation of arenes. Thus, in the last decade, several groups made an effort to develop bifunctional heterogeneous catalysts able to perform C–O hydrogenation of amides where, at least the precious metal performing the H_2_ activation is deposited onto a support (Table 1, entries 5–12). In these bifunctional catalysts the oxophilic metal (Re, Mo, La, Nb, V, or Ti), essential for the C–O selectivity through the activation of the carbonyl group, can be either deposited (Table 1, entries 5–7, 10, and 12) or integrated in the metal oxide type support (Table 1, entries 8, 9, and 11).

The first example of supported bimetallic catalysts, acting at reasonably mild temperatures, was the [PtRe/TiO_2_] catalyst developed by the group of Thompson in 2011 (Table 1, entry 5)^73^. In this work, the active material is formed by XRD invisible Pt particles interacting with Re in such a way that Pt can mediate the Re–O reduction. In addition, the support (TiO_2_) also has an important role related with the activation of C = O bond. Although recycling studies with this catalyst were unsuccessful, two years later Cole-Hamilton and co-workers developed the first continuous flow hydrogenation of amides to amines with it^74^. Around the same time, the group of Breit reported a [PdRe/Graphite] catalyst containing 2–6 nm Pd-based nanoparticles able to hydrogenate secondary and tertiary amides with impressive substrate scope (108 examples, Table 1, entry 6)^25^. In 2017, Kaneda and co-workers published an example of bimetallic supported catalyst performing amide hydrogenation (Table 1, entry 10)^23^, composed by Pt nanoparticles of a mean diameter of 2.2 nm decorated with V and highly dispersed on an inert support such as hydroxyapatite (HAP). Based on X-ray absorption near-edge structure studies, IR, and DFT the authors propose that V in [PtV/HAP] is present as V^5+^ and, in the presence of H_2_, is reduced by Pt to V^3+^, able to activate the amide C = O bonds that Pt species cooperatively hydrogenate. The [PtV/HAP] is unique as it is the first heterogeneous catalyst active for amide hydrogenation, selective towards arene hydrogenation and operating at temperatures and hydrogen pressures as mild as 70 °C to RT and 30 to 1 bar H_2_. More recently, in 2018, Gao and co-workers reported a reusable [Ir/3Mo-KIT-6] catalyst able to hydrogenate *N*-acetylmorpholine to *N*-ethylmorpholine with good selectivity (Table 1, entry 12)^75^.

On the other hand, recently several examples of bifunctional catalysts containing a late transition metal dispersed on a metal oxide support with oxophilic properties have been reported as selective catalysts for the hydrogenation of amides through C–O cleavage. For example, in 2016 the groups of Shen and Shimizu published two materials composed by [Ni/LaAlSiO]^76^ and [Pt/NbO_5_]^77^, respectively, active for the selective hydrogenation of tertiary aliphatic amides (Table 1, entries 8, and 9). In these materials, La and Nb belonging to the supports have a main role activating the carbonyl group. One year later Shimizu et al. also published a [Re/TiO_2_] catalyst for the hydrogenation of secondary and tertiary amides with very good selectivity to amide group vs aromatic rings (Table 1, entry 11)^24^. The selectivity is attributed to the higher affinity of Re towards carboxylic acid derivatives over benzene rings. In general, bifunctional catalysts containing the oxophilic metal integrated at the support require higher temperatures (150–200 °C) to operate efficiently.

Despite the rational design of bifunctional supported catalysts, they only perform efficiently with secondary or tertiary amides, being inactive for primary amides or affording secondary amines as products. The only exceptions to this are the [Rh-MoO_x_/SiO_2_ + CeO_2_] (Table 1, entry 7), developed by Tomishige and co-workers^78^, and the [RuWO_x_/MgAl_2_O_4_] (Table 1, entry 13), more recently reported by De Vos and co-workers^79^. The rhodium/molybdenum catalyst is able to selectively hydrogenate cyclohexanecarboxamide to aminomethylcyclohexane^78^, being crucial the addition of basic CeO_2_ for avoiding the formation of secondary amines. In the case of the ruthenium/tungsten catalyst, the selective hydrogenation of a wider range of aliphatic primary amides is achieved through the utilization of a basic support and, more importantly, NH_3_ partial pressure^79^.

Last year, Kadyrov^80^ reported a different strategy where [Pt/C] catalyzed the selective hydrogenation of tertiary amides through its previous activation as amide acetals or imido esters without isolating the intermediates, but using a two-step procedure.

In 2018, the groups of Milstein and Tomishige reported the first heterogeneous catalysts able to perform efficiently the hydrogenation of amides through C–N cleavage. Milstein et al. developed a [Ag/γ-Al_2_O_3_] catalyst able to hydrogenate mainly secondary amides in the presence of KO*t*-Bu, needed for promoting H_2_ activation and affording good selectivity to C–N cleavage (Table 1, entry 14)^81^. Alternatively, the Tomishige group synthesized and characterized a [Ru/CeO_2_] catalyst active for the hydrogenation of primary amides at mild conditions, using water as solvent and without additives (Table 1, entry 15)^82^. Both catalysts showed reusability and selectivity towards the hydrogenation of arenes. Finally, very recently Sorribes, Andrés and co-workers reported a reusable [Pd/In_2_O_3_] material able to perform the hydrogenation of amides through a C–N cleavage pathway with an improved substrate scope and selectivity in comparison with previous examples (Table 1, entry 16)^83^. Interestingly, the active material is constituted by Pd^2+^ cationic species monoatomically dispersed in the oxide matrix.

## Related catalytic hydrogenative transformations

In the past 10 years, the hydrogenation of amide-related compounds such as imides, ureas, or carbamates has been extensively studied, too. Often similar catalysts have been used, which were originally introduced for the hydrogenation of amides or vice versa. In addition, several interesting reductive transformations that imply the hydrogenation of an amide intermediate as a key step (via C–N or C–O cleavage) have been successfully developed. Many of those methods make use of CO_2_ and carboxylic/carbonic acid derivatives as safe and stable starting materials that, in combination with molecular hydrogen, and amines as external nucleophiles, can give access for a variety of highly valuable chemical compounds^84^. In addition, other more energy-related applications have been studied. The recent developments in this growing area will be briefly discussed in this part of the review.

### Hydrogenation of imides, carbamates, *N*-acyloxazolidinones, and ureas

Imides are closely related to amides, but they are generally more reactive as two carbonyl groups are directly bonded to the N-atom^9,15,16,85^. Thus, owing to the electronic effect of the second carbonyl group, also their hydrogenation is expected to be easier than the one of amides^9,85^. However, the challenge for this class of substrates is the control of the selectivity, as a large variety of possible products can be obtained from imides, for example, diols, amines, amides, isoindolines, isoindolinones, and their alkoxy and amino-substituted analogs, hydroxy lactams, and aliphatic cyclic lactams^85^.

Notably, chiral ω-hydroxycarboxamides and chiral cyclic compounds might be obtained by applying an enantioselective version of this transformation, offering a convenient methodology for the desymmetrization of prochiral substrates (such as glutarimides) and *meso*-cyclic imides. In this sense, having a suitable catalyst and reaction conditions in hand, imide hydrogenation could become a versatile, straightforward, and appealing transformation for the synthesis of different kinds of valuable organic compounds. Consequently, the catalytic reduction of imides has received a lot of consideration in the last years.

**Fig. 4:**
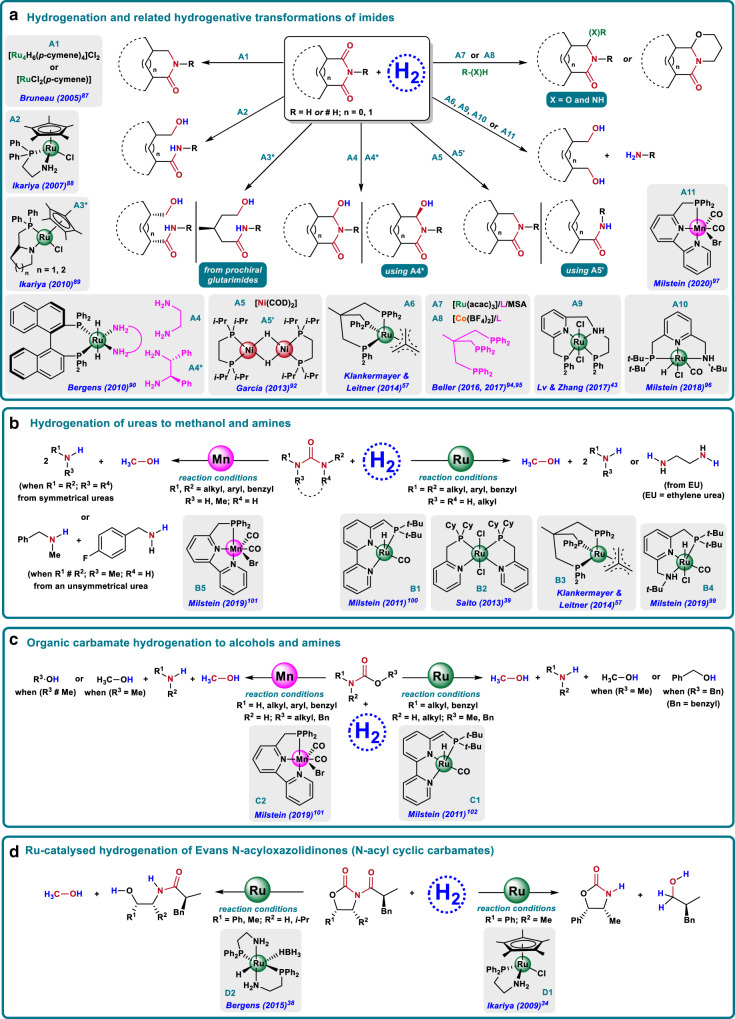
Homogeneously catalyzed hydrogenation of amide-related compounds. **a** Hydrogenative transformations of imides. **b** Ureas to methanol and amines. **c** Organic carbamates to alcohol and amines. **d** *N*-acyloxazolidinones to valuable chemicals.

Early examples dealing with imide hydrogenation involved the use of either heterogeneous Ni catalysts at drastic conditions or in the presence of stoichiometric reagents (i.e., Lawesson’s reagent), or Pd/C in acidic media^85^. The first milder example of reduction of imides using molecular hydrogen dates back from 1993 when Patton and Drago reported the hydrogenation of *N*-methylsuccinimide to *N*-methylpyrrolidinone with different ruthenium homogeneous and heterogeneous systems^86^. In 2005, Bruneau and co-workers published an elegant homogeneous Ru-catalyzed hydrogenation of cyclic imides, succinimides, and phthalimides, via C–O cleavage with a concomitant aromatic ring hydrogenation to afford cyclic aliphatic lactams (Fig. 4A1)^87^. Two years later, Ikariya and co-workers presented the first example of C–N bond hydrogenolysis of cyclic imides to afford ω-hydroxycarboxamides (Fig. 4A2)^88^. Interestingly, compared with previous work, undesired aryl ring saturations of phthalimides were totally avoided. In addition, an enantioselective hydrogenation of prochiral glutarimides promoted by a chiral version of the catalyst (Fig. 4A3*), afforded the desired ω-hydroxycarboxamides in good to excellent enantiomeric excesses^88^. In 2010, the same authors further applied this chiral Ru catalyst (A3*) for the enantioselective hydrogenative desymmetrization of bicyclic imides to give functionalised chiral cyclic hydroxycarboxamides in good yields and enantioselectivities^89^. At the same time, Bergen’s group reported the first selective *mono*-hydrogenation of phthalimides to give hydroxy lactams (cyclic hemiaminals) (Fig. 4A4)^90^. In addition, the desymmetrization of *meso*-cyclic imides via an enantioselective version of the protocol was also performed employing a related ruthenium catalyst, bearing a diamine chiral ligand (A4*), that afforded several enantiomerically pure hydroxy lactams in good yields^90,91^.

Later on, García and co-workers developed two Ni catalysts to mediate either the selective phthalimide C–O bond hydrogenation to isoindolinone (Fig. 4A5)^92^, or the formation of benzamide by the decarbonylation/reduction of the former phthalimide (Fig. 4A5′)^92^. In 2014, the groups of Klankermayer & Leitner reported the double C–N hydrogenation of 1*H*-pyrrole-2,5-dione, an unsaturated succinimide, to 1,4-butanediol mediated by a [(Triphos)Ru(TMM)] complex (Fig. 4A6)^57^. The same year, Agbossou-Niedercorn and co-workers developed a heterogeneous [Rh_6_(CO)_6_/Mo(CO)_6_]-catalyzed full hydrogenation of N-unsubstituted cyclic imides to the corresponding aliphatic cyclic amines^93^.

In 2016 and 2017, Beller’s group published two methodologies employing ruthenium- and cobalt-based homogeneous systems (Fig. 4A7 and A8, respectively) for the reductive alkoxylation/amination of cyclic imides in its inter- and intramolecular versions^94,95^. Both catalytic systems used Triphos as a crucial ligand and completely avoided the phthalimide aryl ring hydrogenation and the (over)reduction of the second carbonyl group. For the first time, the reductive functionalization of one carbonyl group was performed with significant regioselectivity for aryl ring substituted phthalimides.

More recently, Lv’s & Zhang’s (Fig. 4A9)^43^ and Milstein’s (Fig. 4A10)^96^ groups reported two Ru catalysts able to perform the double C–N bond cleavage of cyclic imides. In the case of Lv’s & Zhang's example they use a [Ru-PNNP] complex with no additives, whereas Milstein’s example employs a [Ru-NNP] pincer complex in the presence of catalytic KO*t*-Bu, but achieving a much wider substrate scope. Finally, in 2020 the group of Milstein reported the same reaction catalyzed by a [Mn-NNP] complex in the presence of catalytic KO*t*-Bu (Fig. 4A11)^97^.

Apart from all the progresses made in the area of imide hydrogenation, catalytic hydrogenations of more challenging carbonic acid derivatives such as organic ureas and carbamates have also been studied, albeit to a much lesser extent^9,15,16^. Compared with amides/imides reactivity, these kind of compounds present lower electrophilicity, and therefore much less reactivity as a result of resonance effects of the additional nitrogen, for ureas, or oxygen atom, for carbamates^9^. Despite the difficulties, there is a great interest in their hydrogenation as they are considered direct CO_2_ derivatives, meaning that its hydrogenation to useful products such as MeOH constitutes a way of CO_2_ fixation^98^. With regard to urea derivatives, a small number of homogeneous catalyst based on ruthenium^39,57,99,100^ and more recently on manganese^101^, have been successfully reported (Fig. 4b). In 2011, Milstein’s group published the first example where urea derivatives could be catalytically hydrogenated^100^ using a dearomatizated bipyridine-based ruthenium PNN pincer complex at 110 °C (Fig. 4B1). Then, Saito’s (2013)^39^, Leitner’s & Klankermayer’s group (2014)^57^ and Milstein’s (2019)^99^ groups reported their [Ru-PN], [Ru-Triphos], and [Ru-PNN] complexes for urea hydrogenations (Fig. 4B2–4). Finally, in 2019 Milstein’s group developed the first non-precious metal catalyst able to perform urea hydrogenation based on a Mn-[PNN] complex (Fig. 4B5)^101^.

In addition, the hydrogenation of organic carbamates was also successfully performed using related homogenous metal catalysts (Fig. 4c, d)^101,102^. As an inspiring contribution, in 2011 Milstein and co-workers showed that the bipyridine-based [Ru-PNN] pincer complex, is active for the direct hydrogenation of 2° and 3° organic carbamates to methanol (or other alcohols) and the corresponding amines (Fig. 4C1)^102^. Very recently, the same group applied the first manganese complex for the hydrogenation of a wide range of primary and secondary organic carbamates, albeit inactive for tertiary carbamates (Fig. 4C2)^101^. Complementarily to Milstein work, Ikariya’s group in 2009^34^, and Bergen’s group in 2015^38^, developed two different ruthenium catalyzed hydrogenative protocols that allowed converting Evans-type *N*-acyloxazolidinones to give several interesting hydrogenated chiral products (Fig. 4D1–2).

### CO_2_ hydrogenation to methanol by C–N cleavage of formamides

**Fig. 5:**
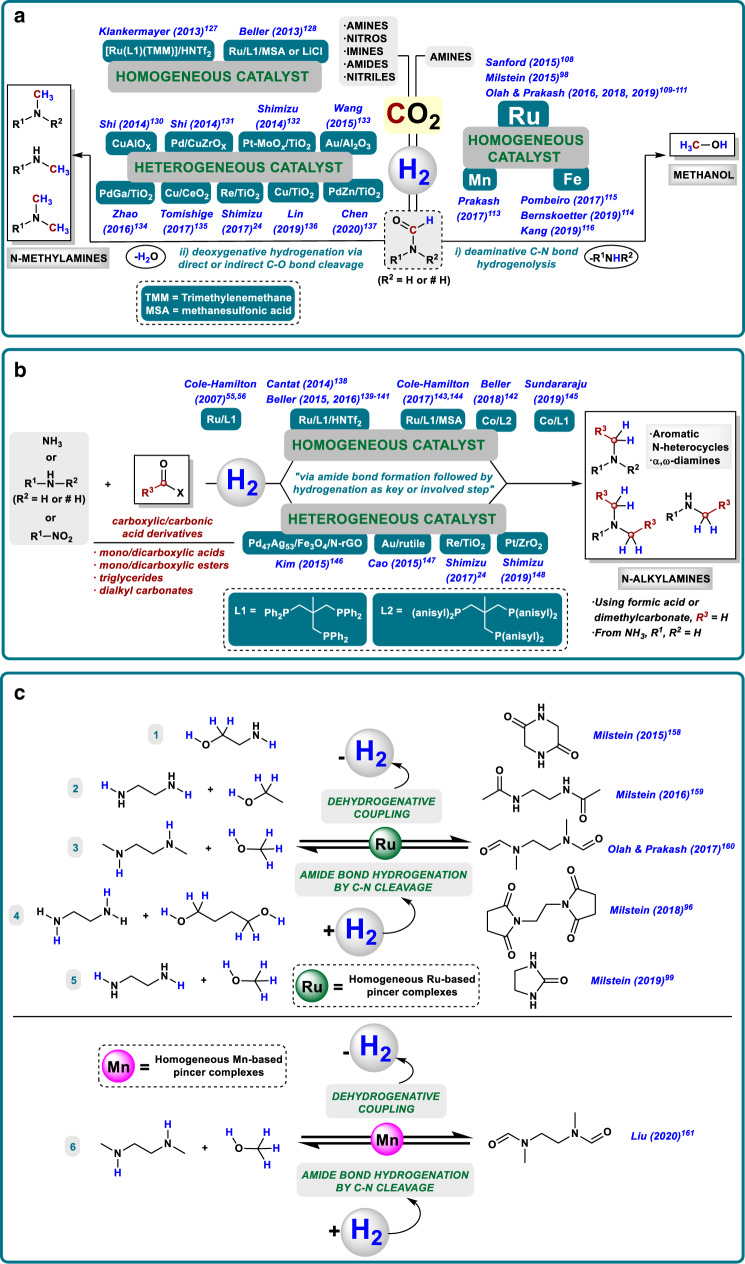
Catalytic reductive processes involving hydrogenation of amides or related derivatives as key step. **a** Amine-assisted CO_2_ hydrogenation to methanol via C–N cleavage (i) and N-methylation with CO_2_/H_2_ through hydrogenative C–O cleavage (ii) of the in situ generated formamide-type compound as key step. **b** Hydrogenative N-alkylation with carboxylic/carbonic acid derivatives. **c** LOHCs systems based on amide-type bonds.

Methanol is among the top five most important basic chemicals^103^ and an essential C1 building block, used as fuel additive and raw material for the production of formaldehyde, formic and acetic acids, dimethylether (DME), olefins, and others. Notably, in the past decade several methanol-to-olefin, and methanol-to-gasoline plants have been implemented in industry on million-ton scale. For the future, it is also considered a promising H_2_-storage material (12.5 wt% H_2_) and a drop-in liquid fuel^104^. The underlying concept of such methanol economy where MeOH would be the central carbon and energy feedstock in a sustainable energy economy was already proposed in the 1990’s by Olah and co-workers^105^. To realize this concept in a sustainable manner, technologies that enable efficient MeOH synthesis from CO_2_ need to be developed in substitution to the current synthesis of methanol from fossil fuels. Ideally, these strategies should be energy efficient and coupled with carbon capture and recycling approaches, which are frequently based on the capacity of amine type compounds of adsorbing CO_2_ in the form of carbamate-type intermediates^106,107^. Hence, a very interesting strategy recently developed consists on the synthesis of methanol from CO_2_ in the presence of amines and H_2_. In this approach, there is a first step of CO_2_ capture to afford a carbamate-type intermediate (normally an alkylammoniun formate salt), which undergoes hydrogenation to give the corresponding formamide. Finally, the in situ C–N hydrogenolysis of the formamide gives methanol and the initial amine, which can be recycled. Indeed, the catalytic systems explored in the search of an effective MeOH from CO_2_ process are mainly metal-ligand cooperative systems^33^, similar to the ones reported for the C–N type hydrogenation of amides (Fig. 5a, i).

Following this general idea, the groups of Sanford^108^ and Milstein^98^ developed in 2015 the first multi-step protocols where MeOH could be obtained from CO_2_ through the C–N hydrogenation of an amide-type compound. Both procedures used a Ru–pincer complex in the presence of catalytic amounts of base. Sanford’s strategy employed [Ru-MACHO-BH] and dimethylamine to give dimethylammonium dimethylcarbamate as intermediate that generates a mixture of DMF/MeOH in a one-pot procedure using a temperature ramp. In Milstein’s approach, a [Ru-PNN] complex is the catalyst and CO_2_ is captured through the use of amino alcohols that afford oxazolidones as intermediates. The latter procedure works without isolation of the oxazolidone, albeit two different steps are required to avoid catalyst deactivation in the presence of CO_2_. From 2016, the group of Prakash and Olah has been working deeply and intensively in improving the one-pot strategy^109–111^. As a result of their efforts, a catalyst TON up to 9900 was reached employing [Ru-MACHO-BH] and pentaethylenehexamine (PEHA) in a biphasic system suitable to be reused four times. In addition, Prakash and co-workers developed a protocol where solid supported amines could be employed as reusable carbon capture systems^112^.

Owing to the increasing interest in replacing precious metals by base metals, and taking into account the opportunity that metal-ligand cooperation affords in this direction, the groups of Prakash^113^ and Bernskoetter^114^ reported two Mn and Fe [PNP] complexes, respectively, for the MeOH synthesis from CO_2_ through formamide hydrogenation. Unfortunately, both catalytic systems deactivated in the one-pot procedure. In addition, Pombeiro and co-workers^115^ as well as Kang’s group^116^ published two iron complexes able to catalyze the synthesis of methanol from CO_2_ in the presence of amines in one-pot fashion. Pombeiro’s system was an iron scorpionate complex working in the presence of PEHA, whereas Kang’s catalyst was an iron tetraphos complex that reduced electrochemically CO_2_ to methanol via diethylformamide.

Notably, most of the reported catalyst systems for hydrogenation of carbon dioxide are deactivated in the presence of even trace amounts of CO. Despite this problem, very recently Prakash and co-workers realized a [Ru-MACHO-BH] catalyzed hydrogenation of CO to methanol via formamide hydrogenolysis with a TON of 539 after 7 days^117^. Independently, Beller and co-workers reported a molecularly defined manganese catalyst for the hydrogenation of carbon monoxide to methanol at 120–150 °C and 50 bar of pressure (TON up to 3170)^118^.

### N-methylation with CO_2_/H_2_

In the above shown conversion of carbon dioxide to methanol process, the selective hydrogenolysis of the in situ generated formamide is crucial. In this key step, methanol and the initial amine are formed as shown in Fig. 1a. In contrast, reactions based on the C–O cleavage pathway will result in the corresponding *N*-methyl amines. Such products are privileged scaffolds present in a variety of bioactive compounds and can be used as versatile intermediates in organic synthesis. Traditionally, N-alkylating methodologies imply the use of toxic reagents or are based on reductive methods employing hydrosilanes or hydroboranes, which suffer from low atom efficiency and tedious work-up procedures^119^. Therefore, the development of more sustainable protocols for their synthesis is highly desired^84^. In this respect, the *N*-methylation of amines using CO_2_/H_2_ mixtures in the presence of a suitable catalyst system is interesting. In this process, the first step is CO_2_ hydrogenation to give in situ the respective formamide, followed by the deoxygenative hydrogenation through direct or indirect C–O cleavage (Fig. 5a, ii). Such reductive transformation gives direct access to *N*-methylamines in one-step procedure, using available CO_2_ and H_2_ as only reagents apart from the amine.

More than two decades ago, Baiker et al. reported for the first time, the synthesis of *N*-methylamines from NH_3_/CO_2_/H_2_ gas mixtures using several metal oxide based heterogeneous catalysts at harsh conditions^120–124^. After these original works, two examples have been recently reported for trimethylamine synthesis from ammonia, or its surrogates, and CO_2_ by Leitner and Klankermayer group with the [Ru/Triphos/Al(OTf)_3_] system^125^, and Shimizu and Toyao et al.^126^ employing a [Pt-MoO*x*/TiO_2_] catalyst.

In addition to the methylation of NH_3_, in the last 6 years, several research groups have contributed to achieve catalytic systems for the *N*-methylation of amines with CO_2_/H_2_ (Fig. 5a, ii)^84^. From the homogeneous side, only two examples have been reported simultaneously by the groups of Klankermayer^127^ and Beller^128^. Both catalytic protocols made use of [Ru/Triphos] complexes in combination with suitable additives (HNTf_2_, CH_3_SO_3_H, or LiCl) as acid co-catalyst, under similar conditions. Remarkably, Klankermayer et al. further expanded the application of this protocol using as starting materials an amide^127^ and several imines^129^, instead of amines.

On the heterogeneous area, more examples have been disclosed, which is easily understood considering the preferred selectivity of heterogeneous catalysts for the C–O hydrogenation of amides. Thus, from 2014 on, several contributions were reported by the groups of Shi, Shimizu & Toyao, Wang & Su, Lin, Yu, Cheng & Zhao and Tamura & Tomishige. In this context, [CuAlO*x*]^130^, [Pd/CuZrO*x*]^131^, [Pt-MoO*x*/TiO_2_]^132^, [Au/Al_2_O_3_-VS]^133^, [PdGa/TiO_2_]^134^, [Cu/CeO_2_]^135^, [Re/TiO_2_]^24^, [Cu/TiO_2_]^136^, and [Pd-ZnO/TiO_2_]^137^ systems were presented as active heterogeneous materials, at different reaction conditions (temperatures from 140 to 230 °C and hydrogen pressures from 25 to 70 bar), for the direct synthesis of *N*-methylamines by using CO_2_/H_2_.

### N-alkylation with carboxylic/carbonic acid derivatives and H_2_

Following the same principle applied for the N-methylation of amines using CO_2_/H_2_ mixtures, carboxylic/carbonic acid derivatives in combination with H_2_ can be employed for a more general N-alkylation of amines–including ammonia–(Fig. 5b)^84^. Theoretically, in those hydrogenative processes as a first step an in situ amide formation takes place, followed by its hydrogenation through direct or indirect C–O cleavage. This type of emerging protocols making use of safer, more stable, and highly accessible alkyl sources offer appealing alternatives to more traditional reductive alkylation methods employing toxic agents, carbonyl compounds, or alcohols via hydrogen autotransfer^119^. However, owing to the kinetic and thermodynamic stability of carboxylic acid derivatives, their application in such hydrogenative N-alkylation processes has been scarcely investigated^84^ and its further implementation still remains an actual task. In fact, either from the homogenous or the heterogeneous side, just a few catalysts have been described^84^ and these are, obviously, closely related with amide C–O hydrogenation catalysts vide supra. Thus, the first homogeneous catalyst was reported by Cole-Hamilton et al. in 2007. They performed the hydrogenation of nonanoic acid in the presence of NH_3_ to give a mixture of *N*-octylamines^55,56^. After this example, interesting developments in this area have been made by the groups of Cantat^138^, Beller (2015, 2016, and 2018)^139–142^, Cole-Hamilton (2017)^143,144^, and Sundararaju^145^. In all these cases, the active catalyst employed Triphos (Fig. 5b, L1) or related substituted derivatives (Fig. 5b, L2) as privileged ligands in combination with a ruthenium or cobalt precursor. Notable developments from 2015 to 2019 reached in the field of heterogeneous catalysis were described by the groups of Kim^146^, Cao^147^, Shimizu^24^, and Shimizu & Siddiki^148^. They made use of carboxylic acids, mainly formic acid, and esters as carboxylic acid coupling partners. In those protocols, [Pd_47_Ag_53_/Fe_3_O_4_/N-rGO]^146^, [Au/rutile]^147^, [Re/TiO_2_]^24^, and [Pt/ZrO_2_]^148^ respectively, were showcased as active systems.

### Amide-type derivatives as H_2_-lean compounds in LOHCs systems

Molecular hydrogen is considered one of the most relevant upcoming energy carriers because of its potential production using renewable energy. As a liquid or under pressure, hydrogen presents a high energy density by weight and only produces water after its combustion^149^. However, owing to its low energy density by volume and difficult storage, safety concerns make the use of hydrogen still a challenging task^150–152^. Hence, the development of new practical and safer methods for improving its storage is demanded. Among the different possibilities currently explored for hydrogen storage, the use of covalent bonds in chemical compounds, specifically so-called liquid organic hydrogen carriers (LOHCs), is a promising approach^152–155^. Most studies of LOHCs are based on hydrogenation/dehydrogenation processes of (N-hetero)arenes, with a relatively high heat of hydrogenation as main disadvantage^156,157^. From 2015 on, the groups of Milstein^96,99,158,159^, Olah & Prakash^160^ and Liu^161^ have successfully developed appealing methodologies related with the use of amide bond hydrogenation and formation through dehydrogenation (from alcohols and amines), as the key steps in which LOHCs systems make use of amide-bond containing derivatives as H_2_-lean compounds (Fig. 5c). The development of metal cooperation catalysts able to promote C–N hydrogenation of amides, as well as dehydrogenation reactions has had an important impact in this field. In fact, all the catalytic systems able to promote amide C–N hydrogenation and its reverse dehydrogenative coupling to release hydrogen are Ru–pincer complexes operating by metal-ligand cooperation^96,99,158–160^, except one recent example of Mn pincer complex^161^. More specifically, in these protocols cyclic bis-amides (peptides)^158^, acyclic amides (including acetamide or formamide based compounds)^159–161^, bis-succinimide^96^, and a cyclic urea derivative (ethylene urea, EU)^99^ are applied as H_2_-lean substrates.

## Future perspectives

### Issues to be resolved

Amide hydrogenation continues to be a challenging transformation highly desired for the efficient preparation of diverse amines under mild conditions. In the last years, significant progress has been achieved in this field by using bimetallic catalysts^8^ or the metal-ligand cooperation catalysts^33^, demonstrating that such ambitious reaction can become a useful tool. In fact, the C–O and C–N hydrogenation of a wide range of amides using H_2_ is nowadays possible in the presence of a suitable homogeneous or heterogeneous catalyst. However, despite these advancements, for the practical application of this transformation, some important issues still have to be resolved. For example, in the case of homogeneous catalysts, the high price caused by the metal and/or the ligands prevents industrial usage. To improve the efficiency of the catalyst systems and, thereby, solving this problem, we believe that a better understanding of the detailed reaction mechanism and intermediates is necessary to design not only more active complexes, but also to understand deactivation phenomena. Based on this deeper knowledge, it might be possible to overcome one of the limitations of homogeneous amide hydrogenation catalysts: the hydrogenation of primary amides especially via C–O cleavage^27,30,62–64^, but also by C–N pathway^36,44,47,52^. In addition, the design of homogeneous catalysts active for the selective C–O hydrogenation of amides is also a challenge, giving the preferred tendency to C–N pathway of this kind of systems. An interesting approach, which researchers working in this field just started to explore, is the use of proper deoxygenating agents, such as boron-based Lewis acid additives. Obviously, designing systems with a catalytic version of the deoxygenating agent can be a promising strategy in homogeneously mediated direct C–O hydrogenation of amides. This might also help in the discovery of new types of homogeneous catalysts for amides hydrogenation. Another interesting approach to make a difference in this area is based on the concept of bimetallic catalysts (inspired from heterogeneous catalysis). Naturally, the typical fine tuning/optimization of known catalysts such as metal pincer, Noyori-type, or Triphos-based complexes will lead to (gradually) improved systems.

Comparing homogeneous catalysts with heterogeneous ones, the latter are in general more selective for the C–O hydrogenation of amides. Although the original heterogeneous catalysts operate under harsher reaction conditions, the development of bimetallic systems allowed for hydrogenation at lower pressure and temperature. A major limitation of heterogeneous materials relies on their low tolerance toward (hetero)aromatic rings or other reducible functionalities. In addition, most supported catalysts are either not able to hydrogenate primary amides so far, or perform hydrogenation of secondary and tertiary amides with very low selectivities. In this respect, the design of novel bifunctional systems offers interesting opportunities. For example, the groups of Kaneda^23^ and Shimizu^24^ recently developed two materials, [Pt/V/HAP] and [Re/TiO_2_], respectively, showing certain tolerance to aromatic ring hydrogenation, which is a positive beginning. Naturally, the rationalization of the real structure of this kind of systems, based on the recent advancements in characterization techniques, as well as the validation of their mechanistic pathway will lead to improved reusable systems active for amides hydrogenation and with hopefully high functional group tolerance.

### Towards practical applications

**Fig. 6:**
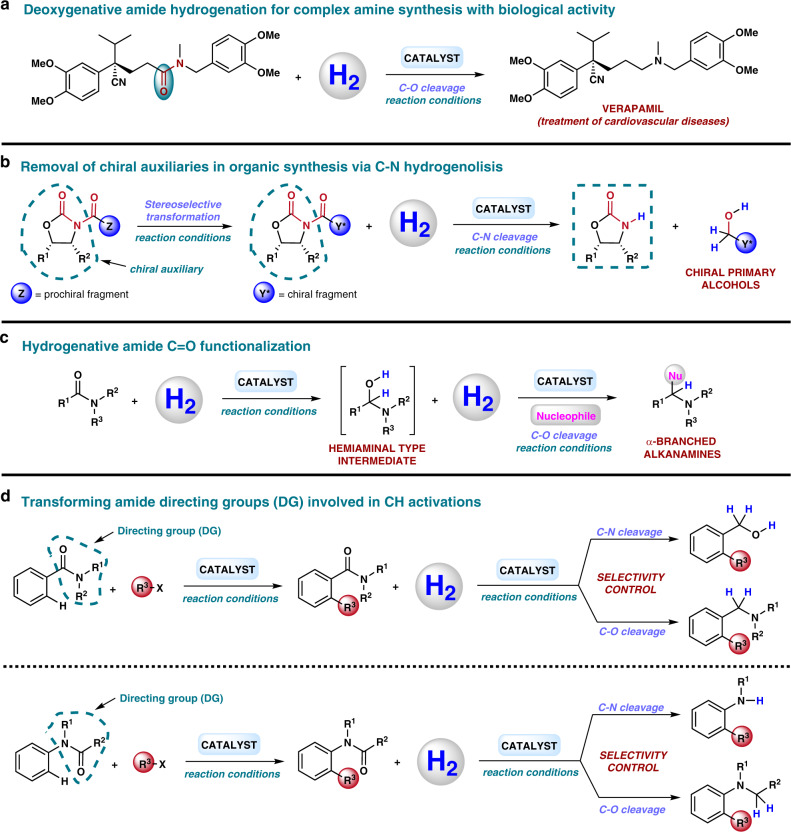
Towards practical applications of amide hydrogenation. **a** Verapamil synthesis using a deoxygenative amide hydrogenation as key step. **b** Removal of chiral auxiliaries in organic synthesis via C–N hydrogenolysis. **c** Hydrogenative amide C = O functionalization. **d** Transforming amide directing groups (DG) involved in CH activations.

The development of practical and cost efficient methodologies for the hydrogenation of amides will have a strong impact in the way organic chemists think about creating C–N bonds, which is of importance for numerous synthetic routes. Hence, a realistic reduction of amides with H_2_ would mean a real change in many large scale protocols that employ metal hydrides or hydrosilanes. Indeed, there are also many reported examples in the area of bioactive compounds in which a drug or natural product synthesis involves as a key step an amide reduction^20,162^. For instance, a large-scale synthesis of verapamil, a drug used for the treatment of cardiovascular diseases, is reported with a last step of C–O amide reduction using borane dimethyl sulfide (BH_3_·SMe_2_) in stoichiometric amounts, requiring a tedious work-up procedure (Fig. 6a)^163^. The same is true for hydrogenative N-alkylation protocols of amines using CO_2_ or carboxylic/carbonic acid derivatives. Those practical transformations make use of more accessible and less toxic reagents compared to traditional N-alkylation procedures^84^.

We consider C–N bond amide hydrogenolysis procedures are probably underestimated in organic chemistry and the life science industries. Without a doubt, they could contribute to the development of greener protection/deprotection methodologies. As an example, it is common in the pharmaceutical industry to employ Evans oxazolidinones as chiral auxiliaries, for example, to obtain chiral alcohols by reduction of the corresponding *N*-acyl oxazolidinone derivative^162^. These protocols normally make use of lithium or sodium borohydride generating a huge amount of residues. Therefore, the use of H_2_ in these deprotection reactions would be a significant advantage from a sustainability perspective (Fig. 6b). In fact, Ikariya and co-workers already demonstrated the feasibility of this deprotection with an excellent maintenance of enantiopurity in the formation of a chiral alcohol from a *N*-acyl oxazolidinone using H_2_ and a Ru catalyst (see also Fig. 4D1)^34^.

Interesting reductions of amides, which recently started to be explored, are reductive functionalizations at the carbonyl group (Fig. 6c). These transformations afford α-branched alkanamines from amides through the formation of a hemiaminal-type intermediate. Normally, this reaction works by employing metal hydrides or silanes as reducing agents in combination with an iridium homogeneous catalyst to afford amines α-substituted with nitrile^164^, amide^165^, or alkyl groups^166^. Clearly, performing this kind of reductive functionalization employing H_2_ is a very attractive research line that will be more exploited in the future. In fact, our group has been recently able to perform the reductive alkoxylation/amination of cyclic imides with H_2_ and Triphos-based systems^94,95^. Interestingly, Maes group recently reported an example in which an amide reductive arylation is performed using isopropanol as transfer hydrogenation agent and [Ru_3_(CO)_12_] as catalyst^167^.

In the last years, amides have acquired also relevance as directing groups (DGs) in C–H activation reactions^168^. The direct conversion of inert carbon–hydrogen bonds into C–C or C–X (X = heteroatom) bonds is one of the most relevant topics in current homogeneous catalysis. However, owing to the high inertness of C–H bonds this transformation is very challenging to achieve in an efficient and selective way. For this reason, the employment of DGs consisting on a coordinating moiety acting as an ‘internal ligand’ can direct a metal catalyst to a certain C–H bond in the molecule, allowing its selective cleavage and subsequent functionalization (Fig. 6d). In this context, the required presence of the DG, frequently an amide, after the CH coupling can be a drawback that prevents from using this methodology when a specific compound is sought. That is why offering sustainable ways of transforming amides in interesting compounds, such as amines and/or alcohols, can entail advantages when using CH activation methodologies that require amide-type DGs, broadening the scope of these transformations^42^.

From an industrial viewpoint, amides are also interesting as integral part of the structure of important polymers, e.g., nylon and related polyamides or polyacrylamides. In this context, the development of efficient hydrogenation methods for amides could have an impact in the recycling of such polymers^42^. Furthermore, new types of materials could be obtained from these known polymers through post-polymerization modifications. In fact, the synthesis of high molar mass poly(allyl alcohol) polymers has been recently investigated using polyacrylamide reduction with borohydrides^169^. The development of active catalytic systems to afford the hydrogenative version of this reaction could be one of the most interesting future applications in the amide hydrogenation area.

Finally, it is important to highlight the already commented energy-focused applications based on amides hydrogenation: low temperature (<150 °C) methanol synthesis from CO_2_ and/or CO using amines and H_2_^170^ and amide-type derivatives as LOHC systems^151^. These strategies in which an amide bond hydrogenation has a central role, have already proved their feasibility and, in our opinion, if we proper invest in their improvement can become fundamental tools in our future society.
